# Comparative genomic characterization and antimicrobial resistance of bacteremia-causing *Enterococcus faecium* and *Enterococcus faecalis* in a Chinese hospital

**DOI:** 10.1128/spectrum.00616-26

**Published:** 2026-08-05

**Authors:** Ning Sun, Yong Chen, Xin Wu, Deyu Gao, Peiran Zhu, Qiuyue Wu, Lining Shi, Xinyi Xia

**Affiliations:** 1 Department of Clinical Laboratory, Jinling Hospital, Affiliated Hospital of Medical School, Nanjing University 12581, Nanjing, China; 2 Institute of Clinical Laboratory Science, Jinling Clinical Medical College, Nanjing University of Chinese Medicine 144990, Nanjing, China; 3 State Key Laboratory of Analytical Chemistry for Life Science, Nanjing University 12581, Nanjing, Jiangsu, China; University of Florida College of Dentistry, Gainesville, Florida, USA

**Keywords:** *Enterococcus faecium*, *Enterococcus faecalis*, whole-genome sequencing, comparative genomics, genome evolution

## Abstract

Enterococci are common commensals of the human gut and important opportunistic pathogens, with *Enterococcus faecium* and *Enterococcus faecalis* being the most clinically prevalent species. A significant epidemiological shift has emerged with an increasing clinical burden of *E. faecium*. To compare genomic evolution of *E. faecium* and *E. faecalis*, we performed whole-genome sequencing on 93 *E. faecium* and 32 *E. faecalis* isolates causing bloodstream infections at a single hospital (2022–2024). Analysis of patient demographics revealed that *E. faecium* infections originated from fewer sources than *E. faecalis*, with a higher proportion deriving from intra-abdominal infections. Multilocus sequence typing identified ST78 and ST789 as the predominant sequence types for *E. faecium,* whereas ST16 and ST179 were most common for *E. faecalis*. *E. faecium* carried more antimicrobial resistance genes and putative virulence marker (PVM)-type virulence genes than *E. faecalis*, with vancomycin resistance predominantly mediated by *vanHAX* (33/93, 35.5%) and a single *E. faecalis* isolate also carrying *vanHAX* (1/32, 3.1%); the structurally incomplete *vanHMX* gene cluster was detected in 11 *E. faecium* isolates. Pan-genome analysis indicated a larger core genome in *E. faecalis* compared to *E. faecium*, consistent with greater plasmid replicon diversity in the latter. Intra-host comparisons showed that two *E. faecalis* pairs from the same patient were clonally related, with one isolate acquiring a *vanHAX* plasmid conferring vancomycin resistance. In contrast, *E. faecium* isolates exhibited marked genomic diversity even among clonally related pairs. These findings suggest that *E. faecium* possesses greater genomic plasticity and adaptive potential to the clinical environment.

**IMPORTANCE:**

This study provides a detailed comparison of clinical and genomic features between *Enterococcus faecium* and *Enterococcus faecalis* from the same hospital setting. We show that *E. faecium* isolates, mainly ST78/ST789, carry more antimicrobial resistance genes and a higher number of putative virulence marker (PVM) genes than *E. faecalis*, reflecting their hospital-adapted nature. *E. faecium* also exhibits a smaller core genome and greater diversity of plasmid replicon types, indicating higher genomic plasticity and capacity for horizontal gene transfer. By contrast, *E. faecalis* retains a larger core genome and a set of classical virulence factors, and its within-host isolates are clonally related. These distinct genomic profiles help to understand how the two species adapt to clinical environments and may inform more targeted infection control strategies and resistance surveillance.

## INTRODUCTION

Enterococci are commensal inhabitants of the human gastrointestinal tract and significant opportunistic pathogens, contributing substantially to the burden of healthcare-associated infections (1). These include bloodstream, intra-abdominal, and urinary tract infections, as well as catheter-related infections (1, 2). The global burden is considerable with enterococcal infections linked to an estimated 439,000 deaths in 2019, highlighting their considerable clinical and economic impact (1–3). Of these, vancomycin-resistant enterococci (VRE) are of particular concern, and recent estimates attribute approximately 14,000 deaths to VRE in 2021 (4). In China, recent data involved enterococci as the second most prevalent Gram-positive clinical isolates, following only *Staphylococcus aureus* (5). The successful persistence of enterococci in hospitals is driven by intrinsic antibiotic resistance, tolerance to disinfectants, and environmental resilience (1, 2). Consequently, detailed epidemiological and mechanistic investigation of enterococci is crucial for developing effective strategies to prevent infections and guide appropriate therapy.

Among enterococci, *Enterococcus faecium* and *Enterococcus faecalis* represent the species of greatest clinical concern (1, 2). While *E. faecalis* was historically the predominant isolate, recent years have witnessed a significant epidemiological shift toward the increasing dominance of *E. faecium* in hospital-acquired infections (1, 4, 6–8). A key factor in this shift is the frequent carriage of potent resistance determinants by *E. faecium*, particularly genes conferring vancomycin resistance (8–12). The widespread clinical use of glycopeptide antibiotics exerts a major selective pressure, solidifying vancomycin-resistant *E. faecium* (VREfm) as a formidable nosocomial pathogen and a leading therapeutic challenge (1, 2, 13, 14). The success of *E. faecium* in healthcare settings involves an evolutionary trade-off, where the acquisition of resistance is balanced by genomic adaptations that sustain fitness in the hospital niche (14, 15). Recent reports do not support a reduction or fine-tuning of virulence, as *E. faecium* instead harbors a larger and distinct repertoire of virulence genes, particularly putative virulence marker (PVM)-type markers (16, 17). Despite this understanding, comparative analyses of the epidemiology, antimicrobial resistance profiles, and genomic evolution of *E. faecium* and *E. faecalis* within the same clinical setting remain limited, particularly studies that directly contrast their genomic architecture and content. Furthermore, when multiple isolates of the same species are commonly recovered from a single patient, it remains unclear whether they represent clonal replicates or distinct clones.

To investigate the genomic and clinical distinctions between these two major enterococcal pathogens, we conducted a single-center, retrospective study. Clinical isolates from bacteremia caused by *E. faecium* and *E. faecalis* were collected between 2022 and 2024 and subjected to whole-genome sequencing (WGS). By comparing clinical characteristics, antimicrobial resistance profiles and resistance genes, virulence factors, plasmid replicons, pan-genomes, and within-host genetic diversity, this study aimed to systematically delineate the differences between *E. faecium* and *E. faecalis* in genomic architecture, adaptive strategies, and clinical epidemiology.

## RESULTS

### Abdominal/gastrointestinal infection as the primary source of enterococcal bacteremia

Between 2022 and 2024, 32 *E. faecalis* and 93 *E. faecium* isolates were collected from 30 and 79 hospitalized patients, respectively. Among these, multiple isolates were recovered from two patients with *E. faecalis* bacteremia and 12 patients with *E. faecium* bacteremia. Because sampling intervals or colony morphology alone could not reliably distinguish clonal replicates, and to fully capture within-patient diversity, all collected isolates were treated as independent samples. Patient characteristics are summarized in Table 1. *E. faecium* bacteremia primarily originated from abdominal or gastrointestinal sources (90%), typically following surgery or invasive procedures (see Materials and Methods for criteria for infection source of enterococcal bacteremia). In contrast, *E. faecalis* bacteremia occurred more often in critically ill patients with significant underlying comorbidities. Although an abdominal source was also common for *E. faecalis* (63%), its infection origins were more diverse. Vancomycin use did not differ between the two groups after excluding patients with unknown exposure, though exposure data were missing for 38% of *E. faecium* cases. Notably, over 30% of all episodes were polymicrobial, with co-infection by *Acinetobacter baumannii* complex being most frequent. *E. faecalis* bacteremia was significantly less common than *E. faecium* bacteremia during the study period (32 vs 93 episodes; *P* < 0.001). Although bacteremia due to *E. faecalis* was associated with a higher rate of clinical deterioration (62% vs 48%), patients in this group had significantly higher baseline comorbidities.

**TABLE 1:** Clinical and demographic characteristics of patients with enterococcal bacteremia

Clinical and demographic characteristics	*Enterococcus faecalis* *N* = 30^*a*^	*Enterococcus faecium* *N* = 79^*a*^	*P* value^*b*^	*q*-value^*c*^
Gender			0.3	0.6
Female	7 (23%)	26 (33%)		
Male	23 (77%)	53 (67%)		
Age	57 (43, 69)	54 (42, 67)	0.6	0.7
Diagnosis category			<0.001	<0.001
Circulatory neurological	1 (3%)	0 (0%)		
Digestive	18 (60%)	52 (66%)		
Infectious	3 (10%)	10 (13%)		
Others	0 (0%)	17 (22%)		
Respiratory	1 (3%)	0 (0%)		
Symptomatic	3 (10%)	0 (0%)		
Trauma burn	1 (3%)	0 (0%)		
Urinary	3 (10%)	0 (0%)		
Pneumonia	26 (87%)	7 (9%)	<0.001	<0.001
Diabetes	5 (17%)	14 (18%)	0.9	>0.9
Hypertension	9 (30%)	20 (26%)	0.6	0.7
Renal insufficiency	20 (67%)	19 (24%)	<0.001	<0.001
Solid organ malignancy	6 (20%)	12 (15%)	0.6	0.7
AKI	10 (33%)	26 (33%)	>0.9	>0.9
Respiratory dysfunction	16 (53%)	34 (44%)	0.4	0.6
Biliary disease	20 (67%)	30 (38%)	0.008	0.024
Recent surgery invasive procedure	29 (97%)	63 (81%)	0.038	0.093
Vancomycin use			0.89	0.89
No	17 (57%)	27 (34%)		
Yes	13 (43%)	22 (28%)		
Unknown	0 (0%)	30 (38%)		
Infection type			0.5	0.7
Polymicrobial	10 (33%)	32 (41%)		
Single	20 (67%)	47 (59%)		
Referral	29 (100%)	72 (91%)	0.2	0.4
Clinical outcome			0.2	0.5
Clinically deteriorated	15 (52%)	35 (45%)		
Clinically improved	9 (31%)	35 (45%)		
Death	3 (10%)	2 (3%)		
Unknown	2 (7%)	6 (8%)		
Infection source			0.002	0.008
Abdominal/gastrointestinal	19 (63%)	71 (90%)		
Catheter-related infection	5 (17%)	3 (4%)		
Central nervous system infection	0 (0%)	1 (1%)		
Pulmonary	0 (0%)	1 (1%)		
Unknown	6 (20%)	3 (4%)		

^*a*^ Data are presented as *n* (%) for categorical variables and median (Q1, Q3) for continuous variables.

^*b*^ Pearson’s Chi-squared test; Wilcoxon rank sum test; Fisher’s exact test; for vancomycin use, patients with unknown exposure were excluded from the statistical comparison.

^*c*^ FDR-adjusted q-value (Benjamini-Hochberg method) for multiple comparisons within this table.

### Population structure and genetic diversity of *E. faecalis* and *E. faecium* from clinical isolates

**Fig 1:**
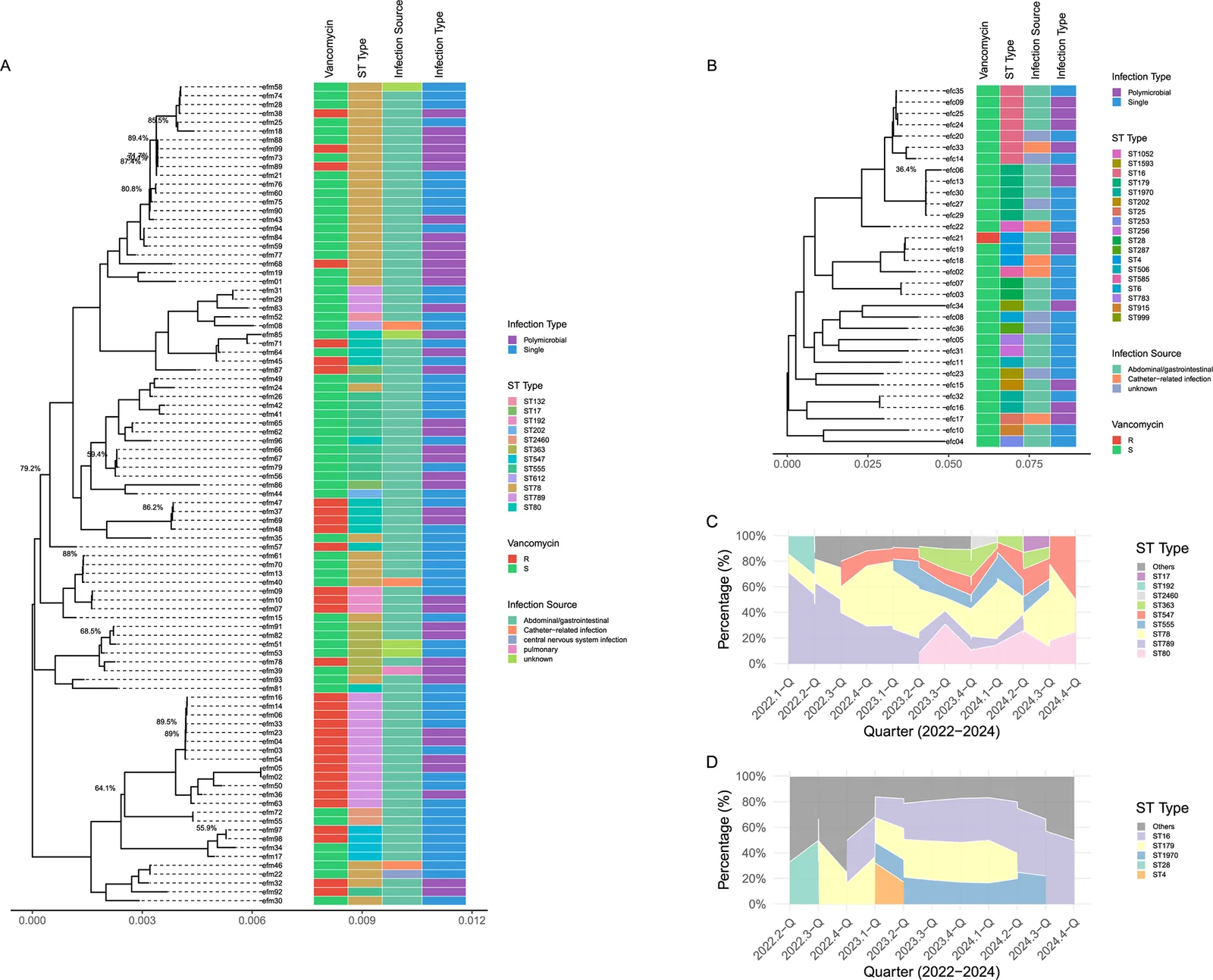
Phylogenetic trees of *E. faecium* (**A**) and *E. faecalis* (**B**) based on core-genome single-nucleotide polymorphisms (SNPs), constructed using FastTree (v2.1.11) under the GTR+Gamma model. Branch support was estimated using SH-like local supports with 1,000 resamples, and only nodes with support <0.90 are shown. Isolates are labeled by ST, infection source, and polymicrobial infection status. Reference genomes (*E. faecium* GCF_009734005.1; *E. faecalis* GCF_000393015.1) were used solely for SNP calling and were excluded from the final phylogenetic trees. The trees were midpoint-rooted using the R package phytools (v2.5-2). Temporal distribution of STs causing bloodstream infections from 2022 to 2024 for *E. faecium* (**C**) and *E. faecalis* (**D**).

Multilocus sequence typing (MLST) analysis revealed divergent population structures between the two species. All *E. faecium* isolates belonged to the hospital-adapted CC17 clonal complex, comprising 12 distinct sequence types (STs). ST78 was the most frequent type (37.6%, 33/93), followed by ST789 (17.2%, 16/93), with ST555 and ST80 each accounting for 11.8% (11/93) (Fig. 1A). Among *E. faecalis* isolates, 18 STs were identified, with ST16 (21.9%, 7/32) and ST179 (15.6%, 5/32) being most prevalent (Fig. 1B). Temporal analysis from 2022 to 2024 revealed distinct dynamics (Fig. 1C and D). In *E. faecium*, ST78 was persistently detected; ST555 and ST80 emerged after 2023, while ST789 appeared intermittently. In *E. faecalis*, only ST16 persisted throughout the study period; all other STs were observed only sporadically.

To further resolve genetic relationships, we performed core genome multilocus sequence typing (cgMLST) and split k-mer analysis (SKA), using thresholds of 20 allelic differences for *E. faecium* (18) and 7 for *E. faecalis* (19), and 7 single-nucleotide polymorphism (SNP) differences for SKA (20). cgMLST identified 4 clusters (≥2 isolates) and 33 singletons in *E. faecium*, including one major cluster of 16 isolates, and 4 clusters (each containing 2 isolates from the same patient) and 24 singletons in *E. faecalis* (Fig. S1). In contrast, SKA assigned a unique group to nearly every isolate of both species (93/93 for *E. faecium*; 31/32 for *E. faecalis*) (Fig. S2). Thus, at the cgMLST level, *E. faecium* showed limited clustering, with only 4 clusters (including one cluster of 16 isolates) and 33 singletons among 93 isolates, suggesting limited clonal expansion confined to a subset of lineages. By contrast, the more stringent SKA threshold resolved nearly all isolates as distinct, indicating high within-species diversity. In *E. faecalis*, both methods revealed mostly singleton patterns, consistent with a more fragmented population structure.

### Antimicrobial resistance profiles of *E. faecium* and *E. faecalis*

**Fig 2:**
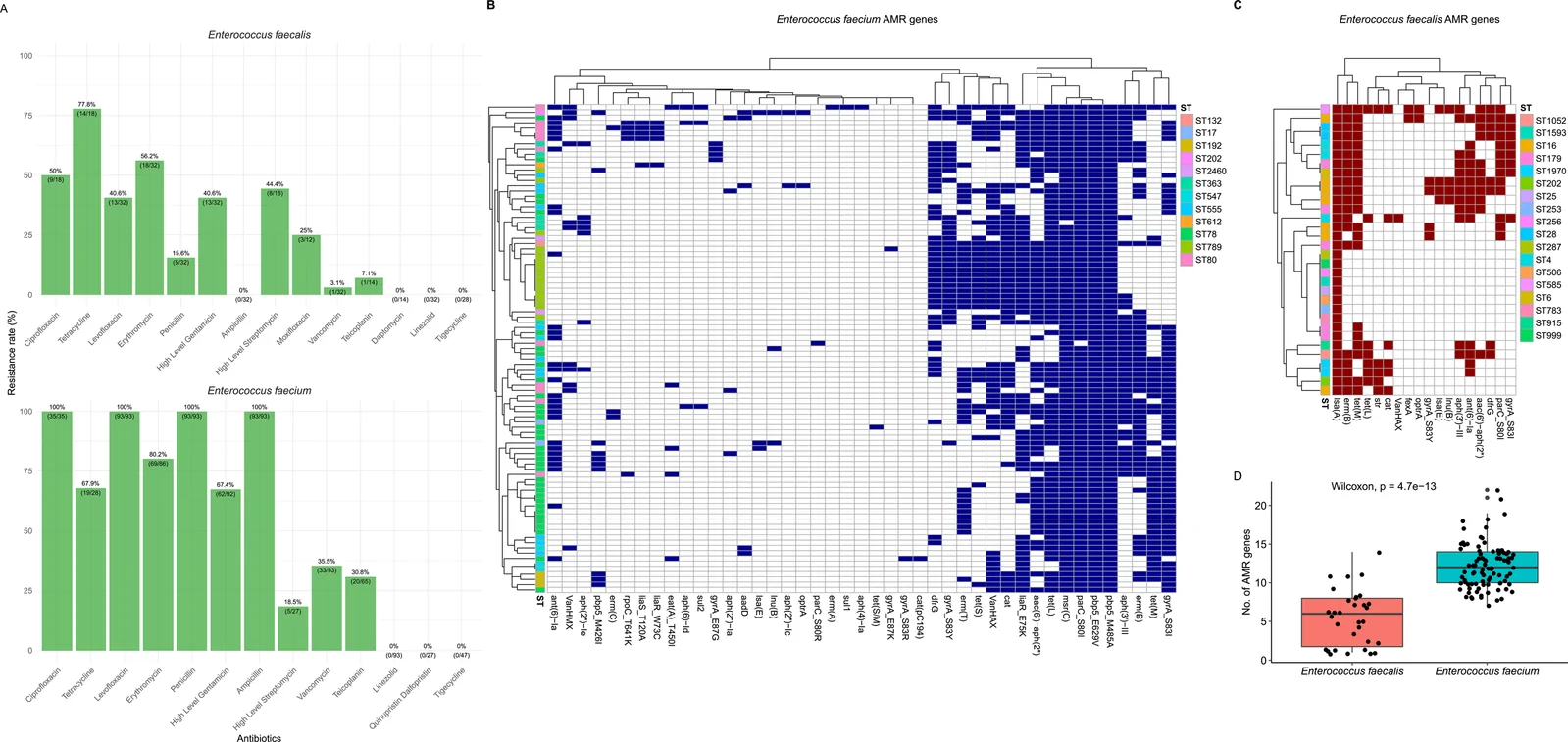
Analysis of antimicrobial resistance. (**A**) Comparative resistance rates of *E. faecium* and *E. faecalis* to selected antibiotics. Numbers above bars indicate the number of isolates tested and the corresponding percentages. *P* values were corrected for multiple comparisons using the Benjamini-Hochberg FDR method; only antibiotics with *q* < 0.05 are indicated as significant. (**B, C**) Heatmaps showing the presence (dark blue) or absence (dark red) of key resistance genes in *E. faecium* (**B**) and *E. faecalis* (**C**). (**D**) Box plot comparing the number of resistance genes per isolate between the two species (*P* = 0.00023, Wilcoxon rank-sum test).

Antimicrobial susceptibility testing revealed that *E. faecium* exhibited significantly higher overall resistance rates than *E. faecalis* (Fig. 2A). All *P* values were adjusted using the false discovery rate (FDR) method of Benjamini and Hochberg (21); significant differences (*q* < 0.05) were observed for ampicillin, penicillin, ciprofloxacin, levofloxacin, erythromycin, high-level gentamicin, and vancomycin. To better relate genotype to phenotype, we combined ResFinder results with AMRFinderPlus-derived point mutations (e.g., in *gyrA*, *parC*, *pbp5*) that are known to mediate resistance in enterococci (Fig. 2B and C). The most prevalent resistance genes in *E. faecium* were *tet(L)* (84.9%) and *aac(6′)-aph(2″)* (66.7%); in *E. faecalis*, *tet(M)* (65.6%) and *erm(B)* (62.5%) predominated. Concordance between genotype and phenotype was calculated as the percentage of isolates in which the genotype prediction (resistant or susceptible) matched the observed phenotype, among all isolates tested for each antibiotic (Fig. S3; Table S3). For several antibiotics, concordance was 100% in both species for ampicillin, ciprofloxacin, and levofloxacin; in *E. faecium* for penicillin; and in *E. faecalis* for vancomycin, teicoplanin, and tetracycline. For linezolid, concordance was high in both species (97.8% in *E. faecium*; 93.8% in *E. faecalis*), with the few discordances being false positives due to *optrA* carriage in the absence of 23S rRNA mutations (22, 23). In contrast, concordance was consistently lower in *E. faecium* than in *E. faecalis* for several antibiotics, including vancomycin (77.4% vs 100%), teicoplanin (73.8% vs 100%), tetracycline (78.6% vs 100%), high-level gentamicin (64.1% vs 87.5%), high-level streptomycin (18.5% vs 72.2%), and tigecycline (25.5% vs 85.7%). Most discordances were false positives (isolates carried resistance genes but remained susceptible), particularly for tigecycline in *E. faecium* (false-positive rate: 35/47, 74.5%), likely due to insufficient expression of *tet(L*) or *tet(M*), or the absence of additional mutations (e.g., in *rpsJ*) required for high-level resistance (24, 25). False negatives were rare, occurring only for penicillin in *E. faecalis* (5/32, 15.6%), possibly reflecting a low-level, non-*PBP5*-mediated mechanism. Overall, *E. faecium* isolates carried a median of 12 resistance genes (range 7–22), compared to only 6 in *E. faecalis* (range 1–14; *P* < 0.001; Fig. 2D).

Vancomycin resistance was observed in 35.5% (33/93) of *E. faecium* isolates, all of which carried the *vanHAX* gene clusters, whereas only one *E. faecalis* isolate (3.1%, 1/32) was resistant and carried the *vanHAX* gene cluster. Among *E. faecium* STs, vancomycin resistance prevalence varied substantially: 100% in ST192 (3/3), 81.3% in ST789 (13/16), 63.6% in ST80 (7/11), 50% in ST547 (2/4), 50% in ST17 (1/2), 16.7% in ST363 (1/6), 14.3% in ST78 (5/35), and 9.1% in ST555 (1/11). For the 21 WGS-positive but susceptible *E. faecium* isolates, *vanA*-specific qPCR performed on multiple colonies was uniformly negative, and the depth of the contigs carrying *vanA* genes was lower than the genome-wide average (Fig. S4; Table S4). To evaluate whether mutations in regulatory genes contributed to the susceptible phenotype, we extracted the *vanR* and *vanS* sequences from all 21 susceptible isolates and compared them with the reference Tn1546 (M97297.1) using BLASTn. Wherever the full coding sequence was retrievable, the nucleotide identity was 100% (Table S5).

**Fig 3:**
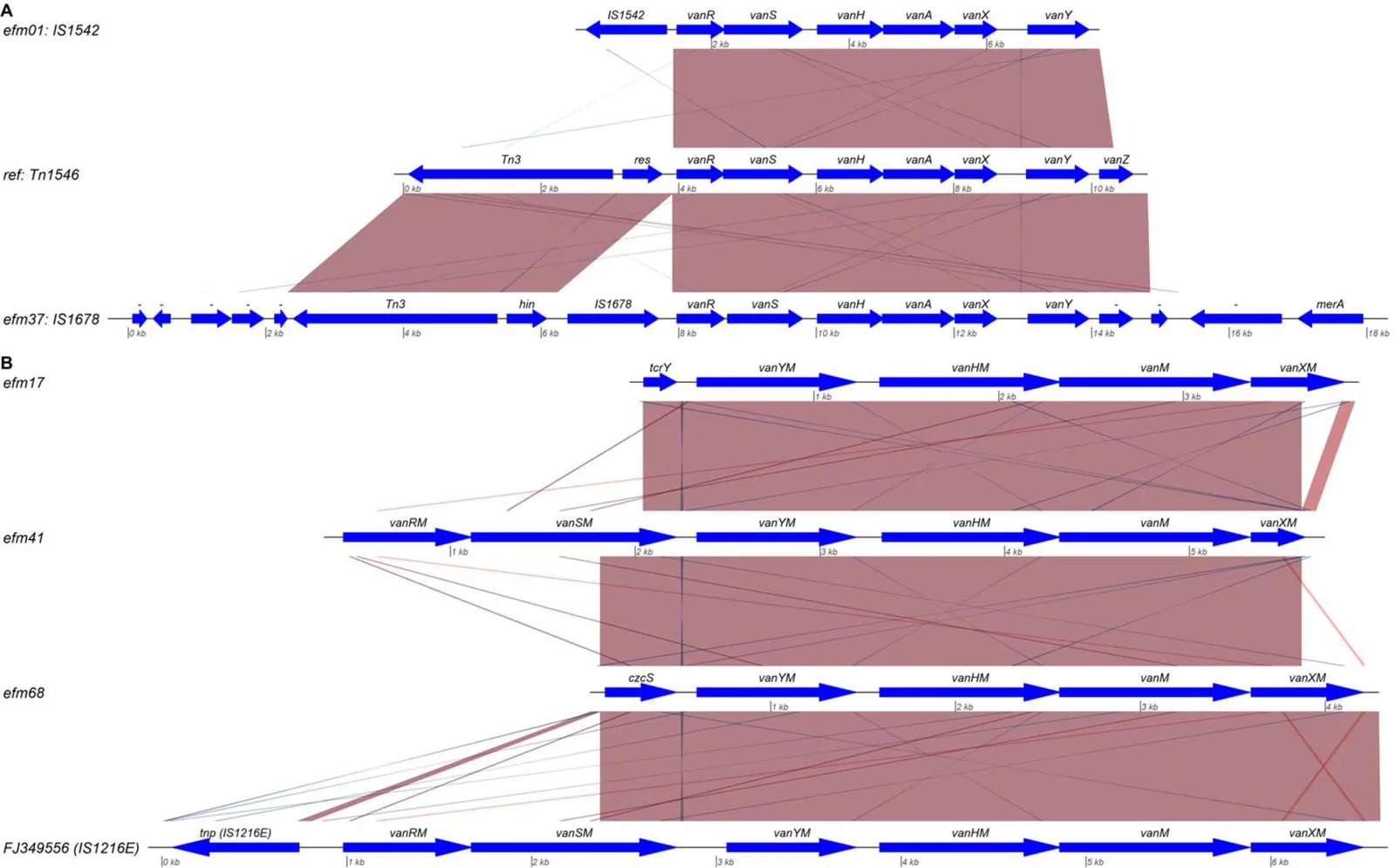
Genetic organization of the *vanHAX* (**A**) and *vanHMX* (**B**) gene clusters. (**A**) Genetic organization of *vanHAX*-carrying contigs compared to the reference transposon Tn1546 (accession: M97297.1). Two variant structures were identified. In isolate efm01, an IS1542 element (IS256 family) is inserted upstream of the regulatory region. In isolate efm37, this position is occupied by an IS1678 element (IS380 family), located downstream of a Tn3-family transposase gene. (**B**) Structure of the *vanHMX* gene cluster compared to the reference transposon FJ349556.1. Isolate efm41 retained the regulatory genes *vanRM/vanSM* but lacked *vanXM*, whereas isolates efm17 and efm68 completely lacked the regulatory genes while preserving the full core resistance genes (*vanYM*, *vanHM*, *vanM*, and *vanXM*). None of the isolates carried an intact IS1216E; only its terminal inverted repeats were detected.

Annotation of contigs containing the *vanA* gene further clarified the genetic context of the *vanHAX* cluster. Unlike the canonical Tn1546 transposon, two distinct arrangements were identified in 17 isolates (Fig. 3A). One configuration comprised a complete *vanHAX* cluster accompanied by IS1542 (IS256 family). The other featured a Tn3 transposase gene upstream of an IS1678 element (IS380 family), followed by the intact *vanA* operon. Among these 17 isolates, the IS1678-associated configuration was more frequent (13/17, 76.5%) and was found exclusively in vancomycin-resistant isolates. The IS1542-associated configuration was less frequent (4/17, 23.5%) and was observed only in susceptible isolates, which carried the complete *vanA* operon yet remained sensitive, likely due to low plasmid copy number or plasmid loss during subculture (Table S4). We detected 11 isolates carrying the *vanHMX* gene cluster (Fig. 3B); 7 of them also carried the *vanHAX* cluster. Compared with the reference transposon (accession: FJ349556.1), seven isolates (including efm41) lacked the *vanXM* gene but retained the regulatory genes *vanRM/vanSM*, whereas the remaining four isolates (including efm17 and efm68) entirely lacked the regulatory genes (*vanRM/vanSM*) but retained the complete core resistance genes (*vanYM*, *vanHM*, *vanM*, and *vanXM*). None of the isolates carried an intact IS1216E; only the terminal inverted repeats of IS1216E were detected.

To place these structural variants in a broader geographic context, we retrieved 553 published genome sequences from the NCBI database (496 carrying *vanHAX* and 57 carrying *vanHMX*, Table S6). After dereplication with CD-HIT at 99.5% identity for *vanHAX* and 100% for *vanHMX*, we extracted 101 contigs harboring *vanHAX* and 55 contigs harboring *vanHMX*. The transposon sequences, defined as the core *van* gene cluster plus 3,000 bp flanking sequences on both sides, were extracted and subjected to phylogenetic analysis. The resulting tree revealed that *vanHAX* genes are highly homologous to epidemic strains from Taiwan (China), Japan, Denmark, and the United Kingdom (Fig. S5A), whereas *vanHMX* genes are predominantly found in Asia, particularly in mainland China (Fig. S5B).

### Virulence gene profiles of *E. faecalis* and *E. faecium*

**Fig 4:**
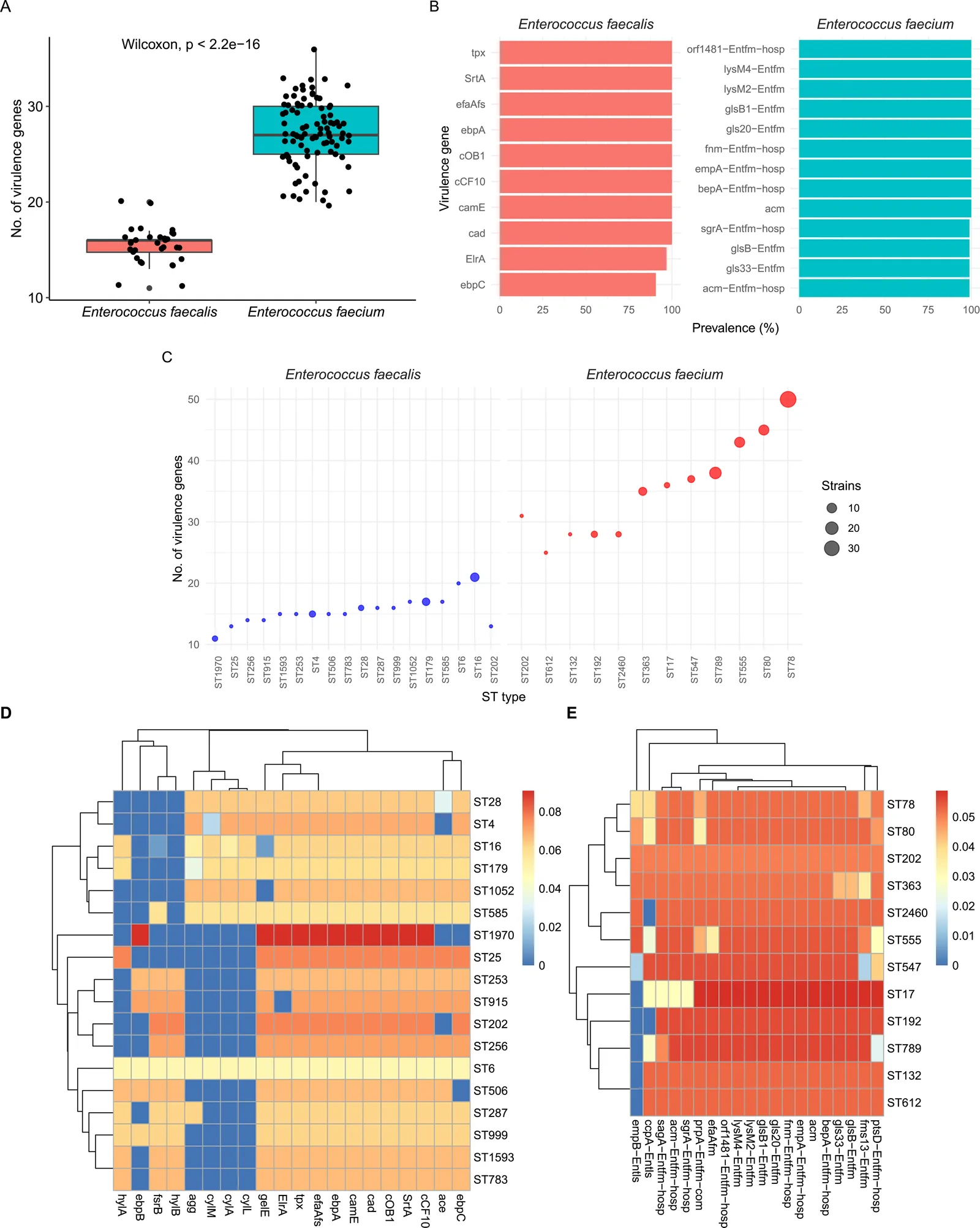
Virulence gene profiles of *E. faecium* and *E. faecalis* based on VirulenceFinder screening against both VFDB and a newly curated PVM database. (**A**) Comparison of virulence gene counts between species (Wilcoxon rank-sum test with Benjamini-Hochberg FDR correction, *P*/*q* < 0.001). (**B**) Prevalence of the ten most common virulence genes in each species. (**C**) Distribution of virulence gene counts across different STs. Heatmaps showing the presence and abundance of virulence genes across STs for *E. faecium* (**D**) and *E. faecalis* (**E**).

Using the VirulenceFinder tool against both the VFDB (https://www.mgc.ac.cn/) and a newly curated database, including PVMs for enterococci (see Table S2) (16, 17), virulence gene profiling revealed a significantly higher burden in *E. faecium* than in *E. faecalis* (median 27 vs 16; Wilcoxon rank-sum test, *P*/*q* = 3.74 × 10⁻¹⁷; Fig. 4A). In *E. faecalis*, eight virulence genes, including *SrtA*, *cCF10*, *cOB1*, *cad*, *camE*, *ebpA*, *efaAfs*, and *tpx*, were detected in all 32 isolates, while *ElrA* (96.9%) and *ebpC* (90.6%) were also highly prevalent. In contrast, *E. faecium* harbored a distinct set of virulence-associated genes. Notably, nine genes, including *acm*, *bepA-Entfm-hosp*, *empA-Entfm-hosp*, *fnm-Entfm-hosp*, *gls20-Entfm*, *glsB1-Entfm*, *lysM2-Entfm*, *lysM4-Entfm*, and *orf1481-Entfm-hosp*, were detected in all 93 isolates (100%). Most of these genes carry the “Entfm” suffix and belong to the PVMs specifically designed for *E. faecium* and *Enterococcus lactis* (16, 17). Among these PVMs, several have been associated with clinical infections and hospital adaptation. According to Freitas et al. (26), Group I markers (*ptsD*, *IS16*, *orf1481*, *sgrA*) are most frequently found in clinical *E. faecium* isolates, whereas Group II markers (*esp*, *ecbA*, *acm*, *hyl*) are more commonly observed in clinical ampicillin-resistant strains. In our collection, *orf1481* and *sgrA* were present in over 98% of *E. faecium* isolates (100% and 98.9%, respectively). *ptsD* and *IS16* were detected in 80.6% and 44.1%, respectively. The *acm* gene was universally present (100%), while *esp* was rare (1.1%). Regarding pilus gene clusters (PGCs), *fms13* (PGC-3) was the most prevalent complete pilus subunit, carried by 87.1% of isolates. Furthermore, these PVMs were almost exclusively enriched in *E. faecium* (median 23 per isolate) and were nearly absent in *E. faecalis* (median 2; Wilcoxon test, *P*/*q* = 0.016), underscoring the species-specific virulence repertoire of hospital-adapted *E. faecium*. Across sequence types, no substantial variation in virulence gene numbers was observed among *E. faecium* STs. Similarly, in *E. faecalis*, the number and variety of virulence genes showed no marked differences across STs (Fig. 4C through E).

### Plasmid replicon profiles of *E. faecium* and *E. faecalis*

**Fig 5:**
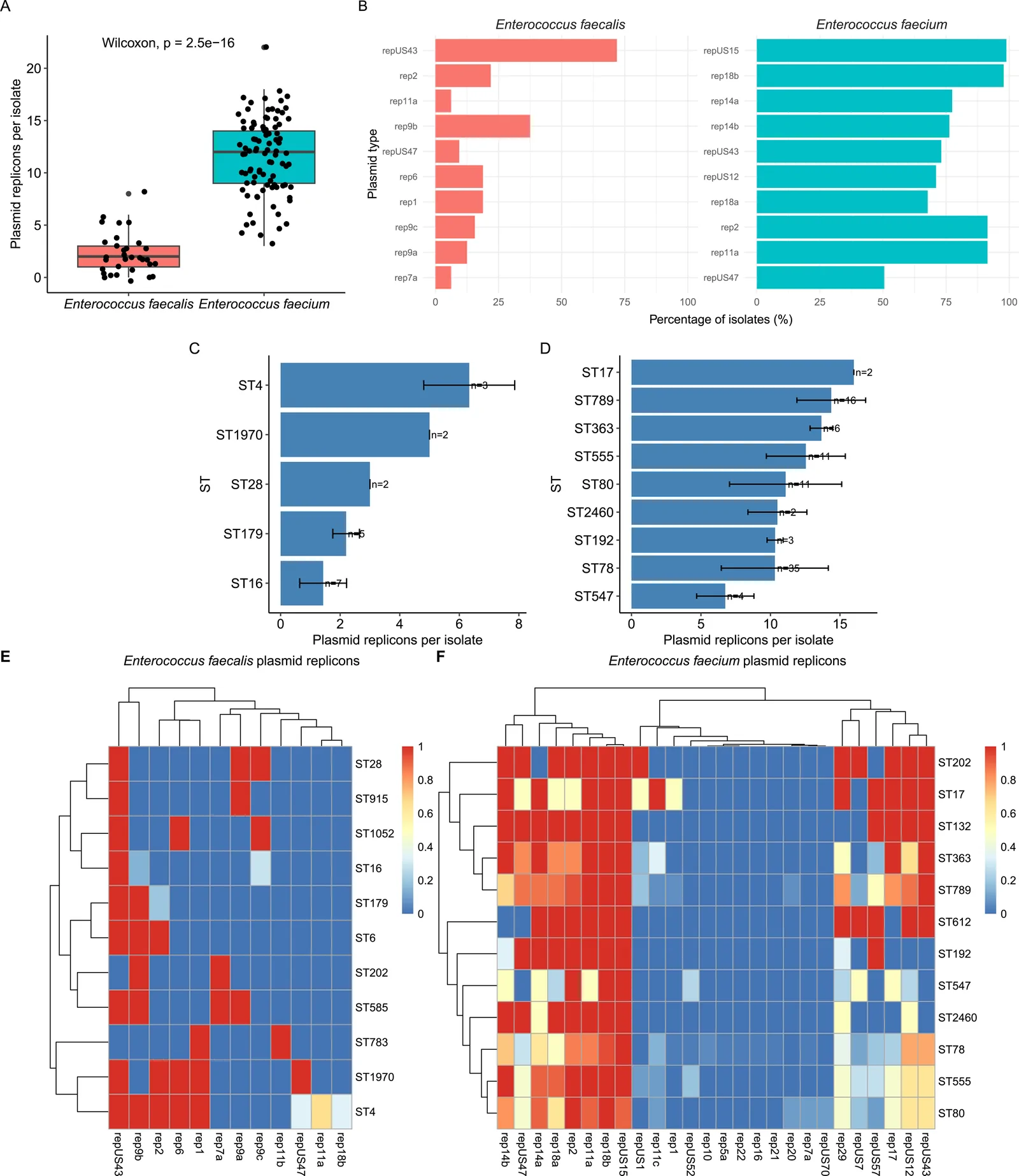
Comparative analysis of plasmid replicons in *E. faecium* and *E. faecalis*. (**A**) Box plot showing the number of plasmid replicon types per isolate for each species (Wilcoxon rank-sum test). (**B**) Prevalence of the ten most common replicon types across isolates. (**C, D**) Mean number of plasmid replicons per ST for *E. faecium* (**C**) and *E. faecalis* (**D**). (**E, F**) Heatmaps depicting the distribution of replicon types across STs for *E. faecalis* (**E**) and *E. faecium* (**F**); colors indicate the proportion of isolates within each ST carrying a given replicon type.

Plasmid prediction analysis indicated that *E. faecium* carried significantly more plasmid replicons than *E. faecalis* (Fig. 5A and B). Across all isolates, the median number of replicons per isolate was 12 (range: 3–22) in *E. faecium* compared with 2 (range: 0–8) in *E. faecalis* (Wilcoxon rank-sum test, *P* < 0.05). A total of 26 distinct replicon types were identified in the 93 *E. faecium* isolates (Fig. 5E), while 12 types were found in the 25 *E. faecalis* isolates that carried replicons (seven *E. faecalis* had no plasmid replicon; Fig. 5F). The predominant replicons differed markedly between species: in *E. faecium*, repUS15 (98.9%; RepA_N family), rep18b (97.8%; Rep_trans family), rep11a (91.4%; Rep3 family), and rep2 (91.4%; Inc18 family) were most common, whereas in *E. faecalis*, repUS43 (71.9%; Rep_trans family) and rep9b (37.5%; RepA_N family) were the most prevalent (Fig. 5B). Distribution across sequence types showed that dominant STs in *E. faecium* generally correlated with higher plasmid carriage, while in *E. faecalis*, the prevalent ST16 was not associated with elevated plasmid loads (Fig. 5C through F).

### Comparative pangenome analysis of *E. faecalis* and *E. faecium*

**Fig 6:**
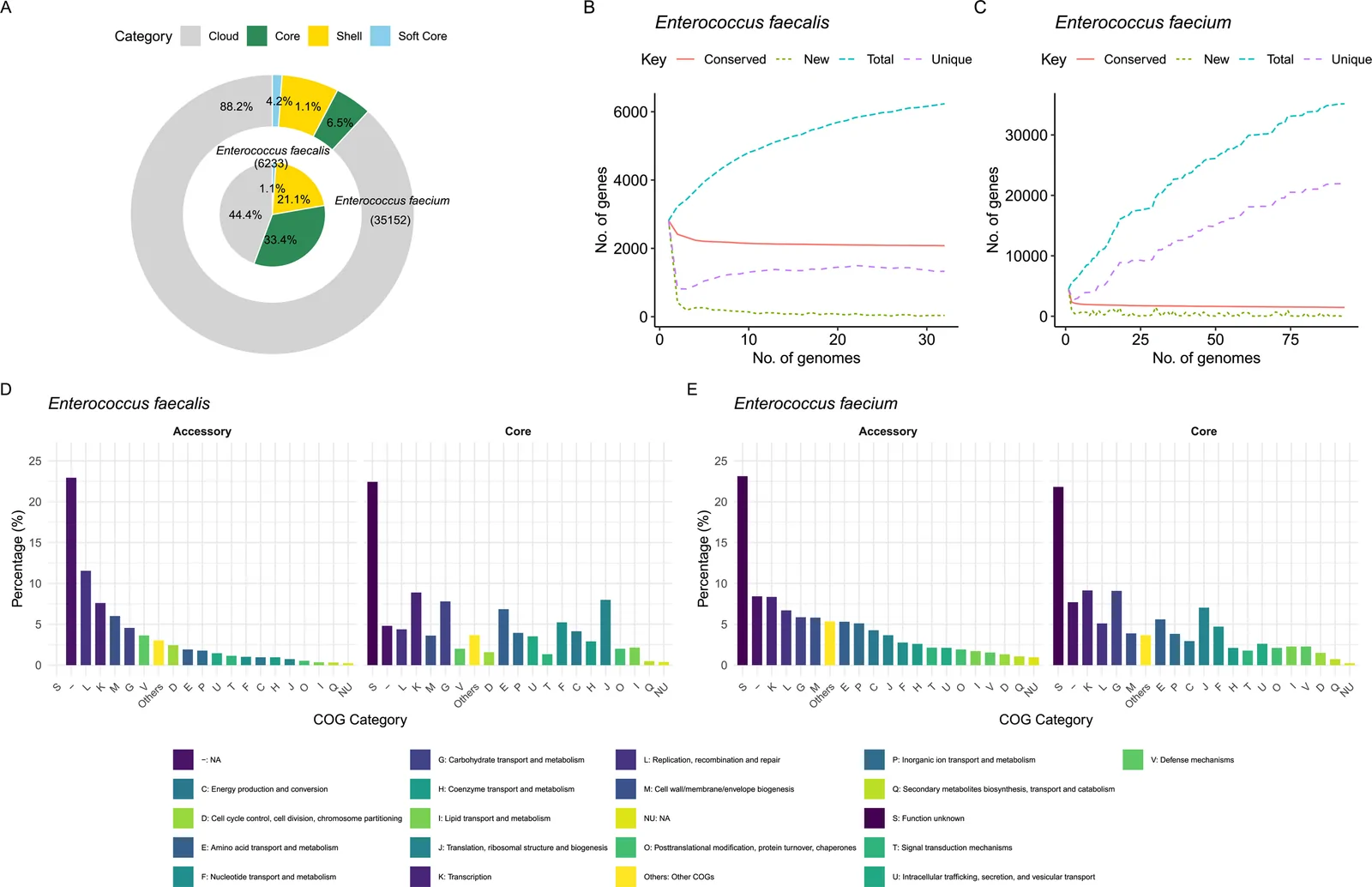
Comparative pangenome analysis of *Enterococcus faecium* and *Enterococcus faecalis*. (**A**) Distribution of gene families. Gene families are categorized based on their prevalence across strains: Core (≥99%), Soft core (≥95% and <99%), Shell (≥15% and <95%), and Cloud (<15%). The total number of gene families in each category is indicated in parentheses. The inner ring represents *E. faecalis* and the outer ring represents *E. faecium*. (**B and C**) Rarefaction curves of *E. faecalis* and *E. faecium*. Dynamics of the total, core (conserved), new, and unique gene families with the sequential addition of genomes. (**D and E**) Functional analysis of core and accessory genes. Gene families were functionally annotated and categorized using eggNOG-mapper (27). The symbol “–” denotes genes that could not be assigned to any COG category.

Pangenome analysis delineated fundamental differences in genomic structure between the two species (Fig. 6). *E. faecium* had a pangenome of 35,152 gene families, with a small core genome (1,470 genes; 4.2%) and a large accessory component. In contrast, *E. faecalis* had a smaller pangenome (6,233 gene families) but a substantially larger core genome (2,079 genes; 33.4%) (Fig. 6A). Rarefaction curve analysis revealed differences in gene repertoire saturation (Fig. 6B and C). For *E. faecalis*, the core genome curve declined sharply before plateauing, and new gene discovery approached zero, indicating a nearly complete sampling of its gene repertoire. In *E. faecium*, the total pangenome expanded rapidly without reaching a plateau, and the unique gene pool increased in parallel, suggesting ongoing gene acquisition and greater genetic plasticity. Functional annotation of the shared core genome highlighted essential cellular processes, including translation, central carbon metabolism, and stress response. Functional categorization using eggNOG-mapper (27) showed no major differences in core gene functions between species (Fig. 6D and E). In the accessory genome, *E. faecalis* exhibited higher proportions of unannotated genes and replication/recombination/repair genes, whereas *E. faecium* showed slightly higher proportions of carbohydrate transport/metabolism and translation/ribosomal structure genes (Fig. 6D and E).

### Genomic features of *E. faecium* and *E. faecalis* within host

**Fig 7:**
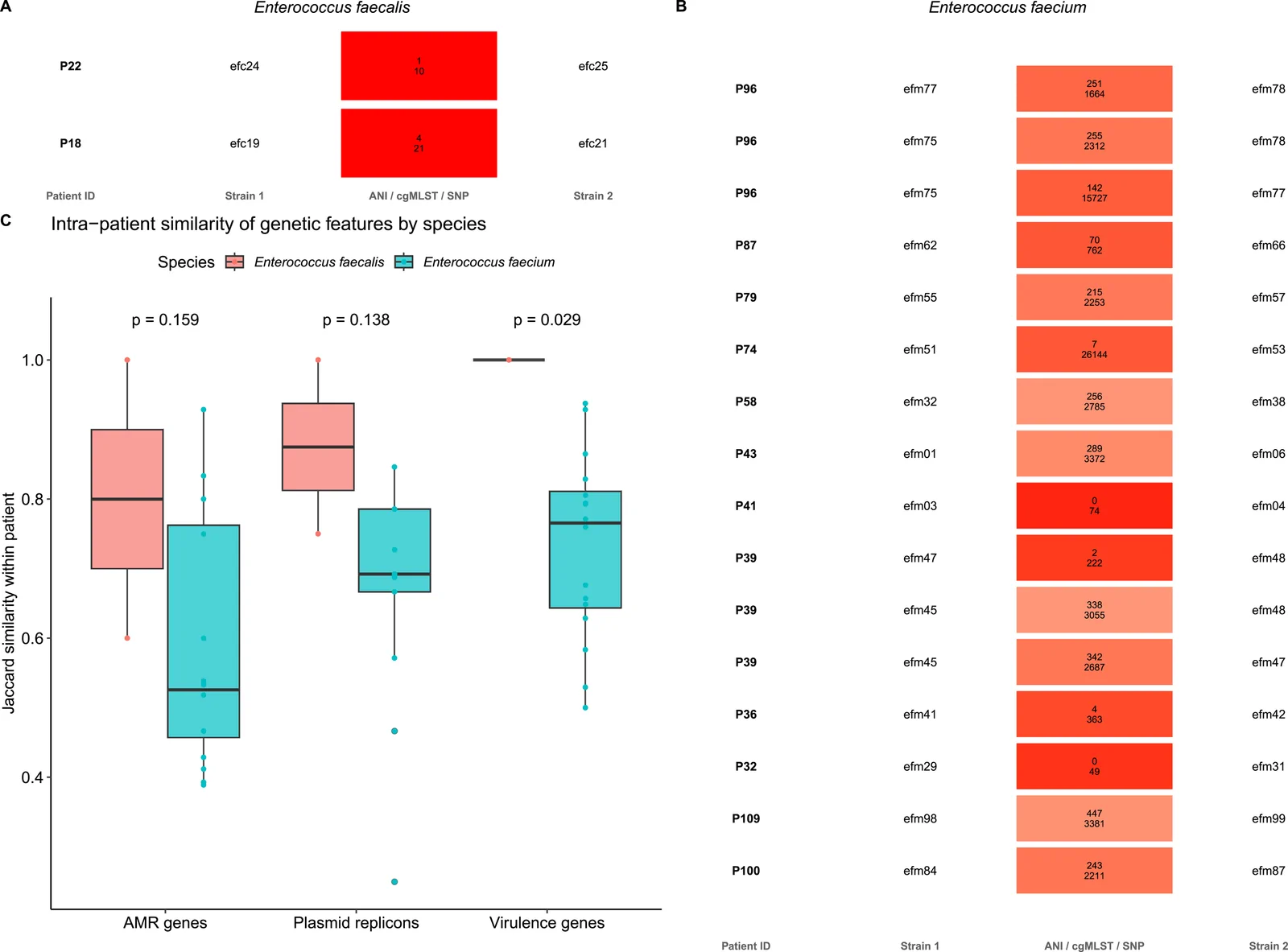
Intra-patient genomic comparison of *E. faecium* and *E. faecalis* isolates. (**A, B**) Pairwise comparisons showing ANI (color), cgMLST allelic distance, and SNP count for *E. faecalis* (**A**) and *E. faecium* (**B**). (**C**) Jaccard similarity of AMR genes, virulence genes, and plasmid replicons among isolates from the same patient (Wilcoxon rank-sum test).

We assessed within-host genomic diversity of isolates from the same patient (Fig. 7). A total of 14 patients yielded 4 *E. faecalis* isolates (2 pairs: 2 patients with 2 isolates each) and 26 *E. faecium* isolates (16 pairs: 10 patients with 2 isolates each and 2 patients with 3 isolates each), for a total of 18 pairs (Fig. 7A and B; Table S1). In *E. faecalis*, both isolate pairs were identified as the same clone (cgMLST distance ≤ 7), with 10 and 21 SNP differences for the two pairs, respectively (Fig. 7A). By contrast, among the 16 *E. faecium* pairs, only 5 (31.2%) were clonally related (cgMLST distance ≤ 20), with SNP counts ranging from 49 to 26,144 and a mean cgMLST distance of 178.8 (Fig. 7B). The remaining 11 pairs represented distinct clones, indicating that some patients harbored more than one *E. faecium* clone. No patient had concurrent or sequential infections with both *E. faecalis* and *E. faecium*. We examined the concordance of AMR genes, virulence genes, and plasmid replicons among isolates from the same patient using Jaccard distances (Fig. 7C). For the two *E. faecalis* pairs, one pair (5 days apart) showed no changes in plasmid replicons, virulence genes, or AMR genes. The other pair (88 days apart) also showed identical virulence genes but marked changes in AMR genes and plasmid replicons, including acquisition of the *vanHAX* cluster that led to vancomycin resistance. For *E. faecium*, even isolates belonging to the same cgMLST cluster displayed substantial variation in AMR genes, plasmid replicons, and virulence genes (Table S2).

## DISCUSSION

Enterococcal bacteremia remains a major clinical challenge, with *E. faecium* and *E. faecalis* dominating the infection spectrum. Although both species share the ability to cause bloodstream infections, their epidemiological trajectories, resistance profiles, and genomic architectures differ substantially. In this study, we performed a comparative genomic analysis of 125 consecutive bloodstream isolates collected from a single tertiary hospital over 3 years (2022–2024), aiming to present the species-specific determinants that drive their success in healthcare settings.

Consistent with recent epidemiological shifts reported in many countries (2, 6–8, 28–31), bacteremia caused by *E. faecium* in our study was approximately three times more frequent than that caused by *E. faecalis*. Both species primarily originated from abdominal or gastrointestinal sources, but *E. faecalis* showed a broader range of infection sources, matching its reputation as a generalist capable of diverse niches (32–34). Our comparative clinical analysis further suggests that differences in patient comorbidities and the nature of invasive procedures may underlie the distinct epidemiological and outcome profiles observed between *E. faecium* and *E. faecalis* bacteremia. Supporting the ecological basis for this divergence, our prior community analysis of biliary and pancreatic fluid samples revealed a greater abundance of *E. faecium* compared to *E. faecalis*, pointing to potential niche-specific adaptations within the gastrointestinal tract that may predispose to different infectious outcomes (35–37).

WGS provided high-resolution phylogenetic delineation of *E. faecium* and *E. faecalis* populations (9, 38, 39). *E. faecium* is broadly divided into two clades (A and B), with clade A encompassing the hospital-adapted, multidrug-resistant CC17 complex characterized by resistance to ampicillin and fluoroquinolones (1). Recent taxonomic work has proposed reclassifying clade B as a separate species, *Enterococcus lactis* (40, 41). All *E. faecium* isolates collected consecutively over the 3-year study period fell within clade A (CC17 complex), with ST78, ST789, ST555, and ST80 being the most common. The dominance of ST78 and the vancomycin-resistant ST80 aligns with their reported prevalence in China (7, 42), contrasting with the major epidemic lineages reported elsewhere, such as ST117 in the United States (43) and several European countries, including Portugal and Germany (44, 45). Notably, ST117 was not detected in our collection. However, a study covering six Chinese provinces did detect ST117 during surveillance in 2023 (7). Thus, the absence of ST117 in our hospital may reflect regional differences in population structure or a recent shift in circulating clones, highlighting the importance of local surveillance.

In contrast to the hospital-adapted clonal structure of *E. faecium*, *E. faecalis* isolates displayed a more diverse population structure. *E. faecalis* isolates were primarily represented by ST16 and ST179, a pattern consistent with reports from other regions (15). These sequence types have been repeatedly recovered from diverse sources, including human clinical samples, animals, and river environments (22, 46). This broader ecological distribution further supports the generalist nature of *E. faecalis*, contrasting with the healthcare specialization of *E. faecium*, all isolates of which belonged to the hospital-adapted clade A (CC17 complex) (7, 10, 16, 17).

Resistance profiles diverged markedly between the species (1, 7, 31, 42, 47). All *E. faecium* isolates were resistant to ampicillin, penicillin, ciprofloxacin, and levofloxacin, whereas *E. faecalis* isolates remained uniformly susceptible to ampicillin. Vancomycin resistance was significantly more common in *E. faecium* (35.5% vs 3.1%). All vancomycin-resistant isolates harbored the *vanHAX* gene cluster, with variants distinct from the canonical Tn1546 transposon, similar to rearrangements reported in Taiwan (48), Japan, Denmark, and the United Kingdom (Fig. S5A). A key finding was the strong association between vancomycin resistance and specific *E. faecium* sequence types, with prevalence varying dramatically: low in predominant ST78 (14.3%), but high in ST789 (87.5%) and ST80 (81.8%). The ST80-associated resistance rate is consistent with an outbreak in Guangzhou, China, suggesting clonal dissemination of successful resistant lineages (7). Notably, 21 WGS-positive *E. faecium* isolates were phenotypically susceptible to vancomycin. Their *vanR*/*vanS* genes were 100% identical to the reference Tn1546, but the *vanA* contigs showed lower sequencing depth than the genome-wide average. The uniformly negative *vanA*-specific qPCR suggests that the vancomycin-resistant subpopulation or plasmid was likely lost during subculture. This observation highlights a common limitation of WGS-based resistance prediction and underscores the importance of phenotypic confirmation.

The detection of the *vanM* operon in 11 *E. faecium* isolates, predominantly in structurally incomplete forms, represents one of the few reports of this genotype outside mainland China. Unlike *vanHAX*, which is globally distributed, *vanM* has been predominantly identified in China (7, 49). Recent genomic surveillance revealed that *vanM* is frequently co-carried with *vanA* in hospital wastewater and clinical isolates, often associated with the emerging ST80 lineage (7, 49). Given that most *vanHMX* clusters in our collection lacked regulatory genes (*vanRM*/*vanSM*) or *vanXM*, these structures likely represent defective or partially acquired transposon remnants rather than functional resistance determinants. However, the co-occurrence of *vanHAX* and *vanHMX* in seven isolates raises the possibility of plasmid-mediated co-transfer, a phenomenon increasingly recognized in Chinese VREfm populations (7). While the clinical impact of *vanHMX* carriage remains unclear, its regional prevalence and frequent association with high-risk clones, such as ST80, underscore the need for continued genomic surveillance to monitor its potential spread beyond China.

Both the general VFDB and a dedicated PVM database specifically designed for *E. faecium* and *E. lactis* were used to profile virulence genes across both species. *E. faecium* carried a higher number of virulence-associated genes belonging to PVMs that reflect adaptations to the clinical environment (26). By contrast, *E. faecalis* retained a set of classical adhesion and biofilm-related genes (e.g., *ebpA*, *efaAfs*, *gelE*), consistent with its role as a generalist (15). *E. faecium*, on the other hand, harbored a markedly higher number and diversity of plasmid replicons (1, 14), suggesting a greater capacity for horizontal gene transfer that may facilitate rapid adaptation under antibiotic pressure. These species-level differences in AMR and virulence gene content and plasmid replicon load were further reflected in genome architecture, as pangenome analysis revealed a much larger core genome in *E. faecalis* (33.4%) than in *E. faecium* (4.2%), indicating greater genomic conservation in the former and higher accessory genome plasticity in the latter. Notably, the replicon profiles observed in our collection differed from those previously reported in European clinical isolates (14, 50). For example, rep17/pRUM, rep2/pRE25, rep14/EFNP1, and rep20/pLG1 were reported as the predominant replicon types in European *E. faecium* isolates (14, 50), whereas our collection was dominated by repUS15 and rep18b, suggesting potential geographical variation in plasmid distribution.

Intra-host genomic comparisons further differentiated the two species. However, the number of such pairs in this study was small. Based on cgMLST and SKA analysis, two *E. faecalis* pairs were considered clonally related. For *E. faecium*, most within-patient isolate pairs were not clonally related (cgMLST > 20), and those that were clonally related still showed clear changes in AMR genes, virulence genes, and plasmid replicons. Based on clustering analysis of different isolates from the same patient, we found that for *E. faecium*, the SKA threshold of 7 SNP differences is clearly too stringent (20), which explains why all 93 isolates were resolved as unique clusters. In contrast, cgMLST with a threshold of 20 allelic differences revealed meaningful clustering (18), indicating limited clonal expansion.

This study has several limitations. First, its single-center, retrospective design may limit the generalizability of the findings, particularly the small *E. faecalis* sample size, which may reduce statistical power; therefore, subgroup comparisons are presented descriptively without formal statistical inference, and the findings should be considered exploratory. Second, multiple isolates from the same patient could overrepresent certain clones, potentially skewing population structure estimates. Third, the 3-year sampling window precludes precise reconstruction of transmission chains or estimation of evolutionary rates. Future prospective studies with denser sampling and more complete clinical data are needed to address these questions. Finally, the proposed differences in evolutionary trajectory require validation through additional *in vitro* and *in vivo* assays or population genomic surveys across diverse niches.

In summary, comparative genomic analysis of bacteremia isolates revealed marked differences between *E. faecium* and *E. faecalis. E. faecalis* displayed a more diverse population structure (18 STs from 32 isolates) and a larger core genome, suggesting a broader genetic repertoire without a single dominant clinical lineage. In contrast, *E. faecium* was dominated by a few closely related sequence types belonging to the CC17 complex, and showed a smaller core genome but greater diversity of plasmid replicons, indicating higher genomic plasticity. These differences in population structure and genomic architecture are consistent with adaptive strategies to the healthcare environment.

## MATERIALS AND METHODS

### Isolates collection and identification

All consecutive enterococcal bloodstream isolates recovered from hospitalized patients at Jinling Hospital between January 2022 and December 2024 were collected. A total of 125 isolates were obtained, comprising 93 *E. faecium* (from 79 patients) and 32 *E. faecalis* (from 30 patients). For patient-level clinical analysis (Table 1), only the first isolate per patient was used. For genomic and resistance analyses, if multiple isolates from the same patient were positive within an interval of ≤4 days, only the first isolate was retained; isolates obtained after >4 days were all included. Species were identified by matrix-assisted laser desorption/ionization time-of-flight mass spectrometry (MALDI-TOF MS; Zhongyuan Huiji, China). Antimicrobial susceptibility testing was performed using the VITEK 2 COMPACT system (bioMérieux, France). Because the testing panels varied over the study period, not all antibiotics were tested for every isolate. Disk diffusion was used for confirmation of vancomycin results. Interpretive criteria followed the Clinical and Laboratory Standards Institute guidelines (M100, 2023 edition).

### Criteria for infection source of enterococcal bacteremia

The primary source of bacteremia was determined by a senior infectious disease clinician and a clinical microbiologist based on comprehensive review of electronic medical records, including clinical presentation, imaging findings, surgical reports, records of invasive procedures, and microbiological culture results from other anatomical sites (e.g., peritoneal fluid, bile, urine, respiratory specimens). Source categories were defined as: abdominal/gastrointestinal (including intra-abdominal infection, biliary tract infection, gastrointestinal surgery, or perforation), catheter-related, urinary tract, respiratory, and others. Any disagreements between the two reviewers were resolved by consensus after re-evaluation of the available data.

### DNA extraction and whole-genome sequencing

Bacterial isolates were inoculated onto blood agar plates and incubated at 35°C with 5% CO₂ for 24 h. Single colonies were inoculated into nutrient broth and cultured overnight with shaking. Cells were harvested by centrifugation, and genomic DNA was extracted using an automated nucleic acid extraction system (CWE960, Cowin Biotech, China). DNA libraries were prepared with minor modifications to a previously described protocol (51). DNA concentration and purity were assessed with a NanoDrop 2000 and a Qubit fluorometer (both Thermo Fisher Scientific, USA), respectively. Sequencing libraries were prepared from 100 ng of genomic DNA per sample using the CWsaq Universal DirectFast DNA Library Prep Kit (Cowin Biotech, China) according to the manufacturer’s protocol, which included fragmentation, end repair, adapter ligation, and PCR amplification. Library fragment size was analyzed on an Agilent 5400 system, and concentration was determined by quantitative PCR. Paired-end sequencing (2 × 150 bp) was performed on an Illumina NovaSeq 6000 platform, generating approximately 6 million reads per sample.

### Bioinformatic analysis

The bioinformatic analysis followed an established pipeline with minor modifications (20). Raw sequencing reads underwent quality control and adapter trimming using Fastp (v0.24.0) (52). *De novo* genome assembly was performed with SPAdes (v3.15.5) (53), and assembly quality was assessed using QUAST (v5.2.0, https://github.com/ablab/quast). Potential contamination was screened with Kraken2 (v2.1.2) (54). Contigs shorter than 500 bp or with a sequencing depth below one-tenth of the genome-wide average were filtered out.

MLST was performed using mlst (v2.23.0, https://github.com/tseemann/mlst); non-typical isolates were confirmed via the PubMLST website (https://pubmlst.org/multilocus-sequence-typing). Whole-genome average nucleotide identity (ANI) was computed for all pairs of genomes using fastANI (v1.34) (55) with default parameters. cgMLST was performed using pymlst (v2.3.1) (56) with the scheme available at https://www.cgmlst.org/. Isolates differing by ≤20 alleles were considered clonally related (18–20). Clustering with SKA (split k-mers, v1.0) was performed with a threshold of 7 SNP differences and an identity of 0.9 (20, 57).

Antimicrobial resistance genes were identified with ResFinder (v4.6.0) using the ResFinder database from CGE (https://genepi.food.dtu.dk/resfinder) with default thresholds of 90% nucleotide identity and 60% minimum coverage relative to the reference gene sequence. Daptomycin-associated mutations were identified using AMRfinderPlus (v4.0.23) (58), and linezolid resistance determinants were screened using LRE-Finder (https://cge.food.dtu.dk/services/LRE-finder/). Intrinsic resistance determinants [e.g., *msr(C*), *aac(6′)-Ii*, *pbp5* mutations, *lsa(A*)] are not included in the acquired resistance gene summary. To assess regulatory gene integrity in vancomycin-susceptible but WGS-positive *E. faecium* isolates, *vanR* and *vanS* sequences were extracted from the corresponding contigs and aligned against the reference Tn1546 (M97297.1) using BLASTn (v2.12.0) with default parameters. Virulence factors were screened using the VirulenceFinder (https://cge.food.dtu.dk/services/VirulenceFinder/) against both VFDB database (https://www.mgc.ac.cn/VFs/) and a newly curated PVM database using default parameters (≥80% nucleotide identity and ≥60% minimum coverage). The PVM database was specifically designed for *E. faecium* and *E. lactis* to capture virulence genes associated with hospital adaptation and infection (16, 26, 59). The plasmid replicons were screened using PlasmidFinder (https://cge.food.dtu.dk/services/PlasmidFinder/), which uses a curated database that specifically considers Enterococcus-associated replicons.

For phylogenetic reconstruction, core-genome SNPs were called with snippy (v4.6.0, https://github.com/tseemann/snippy) using *E. faecium* strain SRR24 (Assembly GCF_009734005.1) and *E. faecalis* strain T5 (Assembly GCF_000393015.1) as reference genomes. Recombination regions were removed using Gubbins (60). These reference sequences were used solely for SNP calling and were excluded from the core genome alignment prior to phylogenetic tree inference. A maximum-likelihood tree was inferred from the filtered alignment with FastTree (v2.1.11) under the GTR+Gamma model (61). Branch support was assessed using SH-like local support values computed by FastTree with 1,000 resamples (default), and nodes with support ≥0.90 were considered well-supported. The trees were then rooted at the midpoint using the R package phytools (v2.5-2).

All genomes were uniformly annotated with Prokka (v1.14.6) (62), and a comparative pangenome analysis was conducted using Roary (v3.13.0) (63). To compare the genetic organization of the *vanHAX* and *vanHMX* gene clusters, the corresponding contigs were annotated using Prokka (v1.14.6) (62) for *vanA*-carrying isolates and Bakta (v1.8.2) (64) for *vanM*-carrying isolates. Insertion sequences (IS) were identified by BLAST search against the ISfinder database (https://tncentral.ncc.unesp.br/ISfinder/index.php). Pairwise nucleotide alignments of the transposon regions were performed using BLASTn (v2.12.0). The resulting alignments and gene annotations were imported into R and visualized with the genoPlotR package (v0.8.11) to produce the final gene maps.

### Statistical analysis

Continuous variables are reported as mean ± standard deviation or median (interquartile range), as appropriate. Categorical variables are summarized as counts and percentages. Statistical analyses were performed with R software (version 4.5.2). For comparisons of clinical characteristics, phenotypic resistance rates, virulence gene counts, and plasmid replicon numbers between species, Fisher’s exact test (or chi-square test when expected frequencies ≥5) was used for categorical variables, and the Wilcoxon rank-sum test for continuous variables. When multiple independent comparisons were performed (e.g., antibiotics, virulence genes, or clinical variables), *P* values were adjusted using the Benjamini-Hochberg FDR method. An adjusted q-value < 0.05 was considered statistically significant. For isolated single comparisons (e.g., comparison of median resistance gene numbers between species, or comparison of plasmid replicase counts between species), unadjusted *P* values are reported with a significance threshold of 0.05.

## Data Availability

The raw sequence data reported in this paper have been deposited in the Genome Sequence Archive (accession CRA037327; BioProject PRJCA056215) and are publicly accessible at https://ngdc.cncb.ac.cn/gsa.

## SUPPLEMENTAL MATERIAL

Supplemental figures spectrum.00616-26-s0001.pdf Figures S1 to S5.

Supplemental tables spectrum.00616-26-s0002.xlsx Tables S1 to S6.

ASM does not own the copyrights to Supplemental Material that may be linked to, or accessed through, an article. The authors have granted ASM a non-exclusive, world-wide license to publish the Supplemental Material files. Please contact the corresponding author directly for reuse.
