# Supplementary material for: Comparative genomic characterization and antimicrobial resistance of bacteremia-causing Enterococcus faecium and Enterococcus faecalis in a Chinese hospital
Source: Microbiol Spectr. 2026 Aug 5;14(9):e00616-26. doi: 10.1128/spectrum.00616-26 (PMC13532331; doi:10.1128/spectrum.00616-26)
Supplement: Supplementary file 1 [file spectrum.00616-26-s0001.pdf]

Supplementary Materials

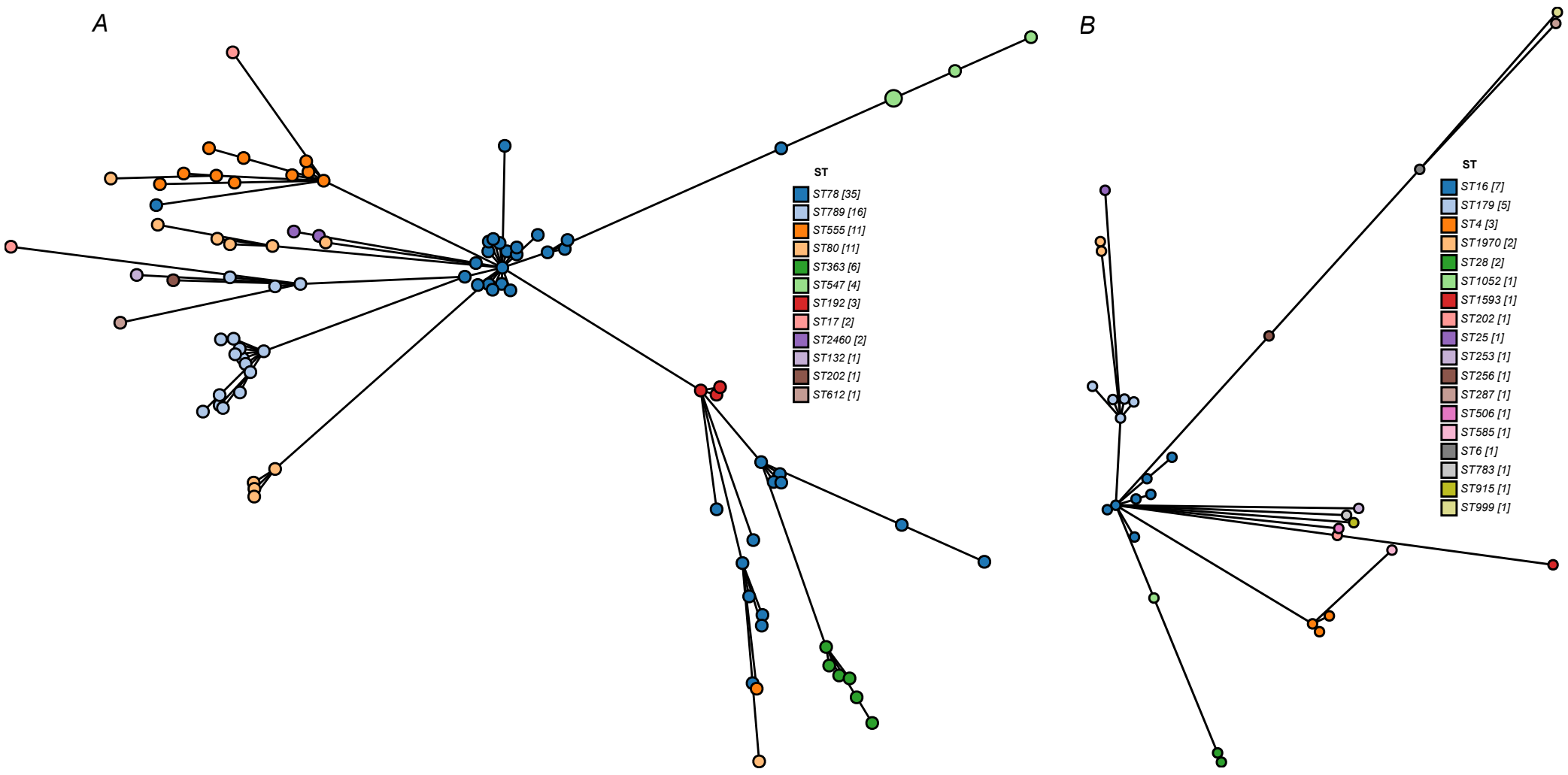

Figure S1. Minimum spanning trees of *E. faecium* (A) and *E. faecalis* (B) based on cgMLST distances, constructed using GrapeTree with the MSTreeV2 algorithm. Sequence types (STs) and their counts are annotated.

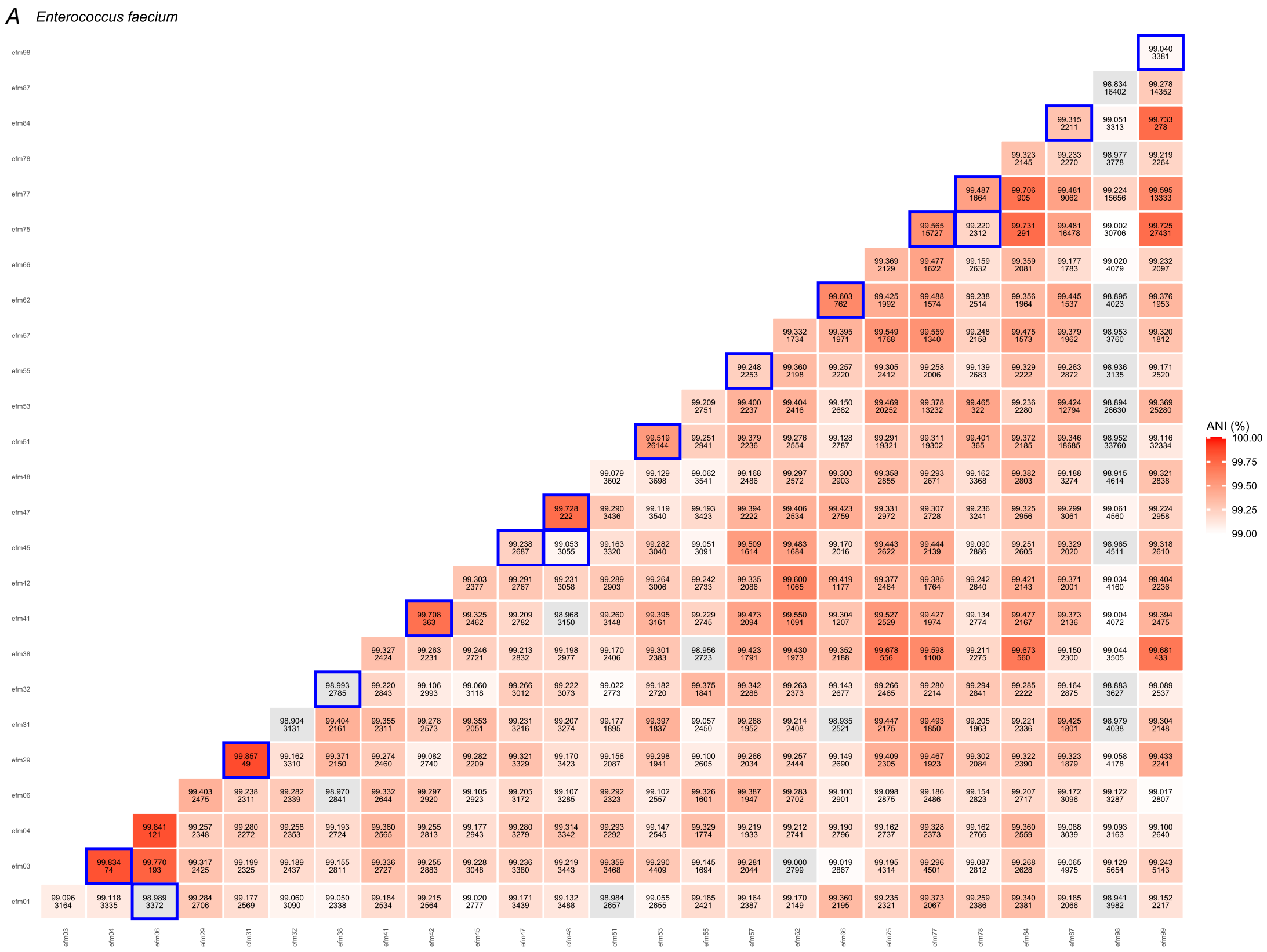

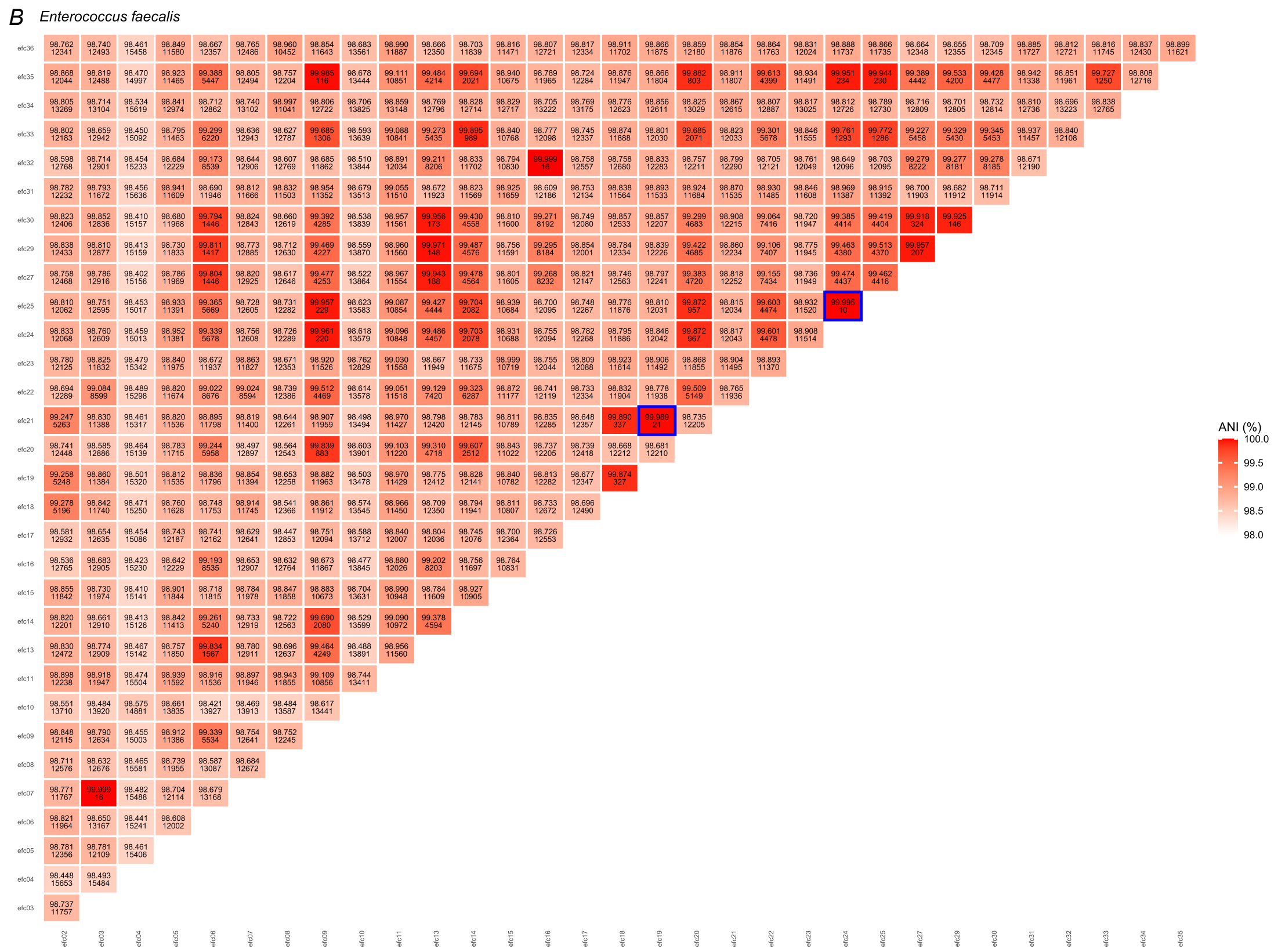

Figure S2. Pairwise genomic comparisons of *E. faecium* and *E. faecalis*. (A) *E. faecium* isolates involved in recurrent infections (blue borders indicate pairs from the same patient). (B) *E. faecalis* isolates (blue borders indicate pairs from the same patient). Each cell represents a comparison between two isolates; color intensity reflects the average nucleotide identity (ANI, %). The numbers inside each cell are formatted as "ANI value / SNP count", where SNP counts were derived from SKA analysis. For *E. faecium*, only isolates from patients with multiple positive cultures are shown. For *E. faecalis*, all isolates are displayed. Blue borders highlight within-patient pairs. ANI values close to 100% and low SNP counts indicate high genomic relatedness.

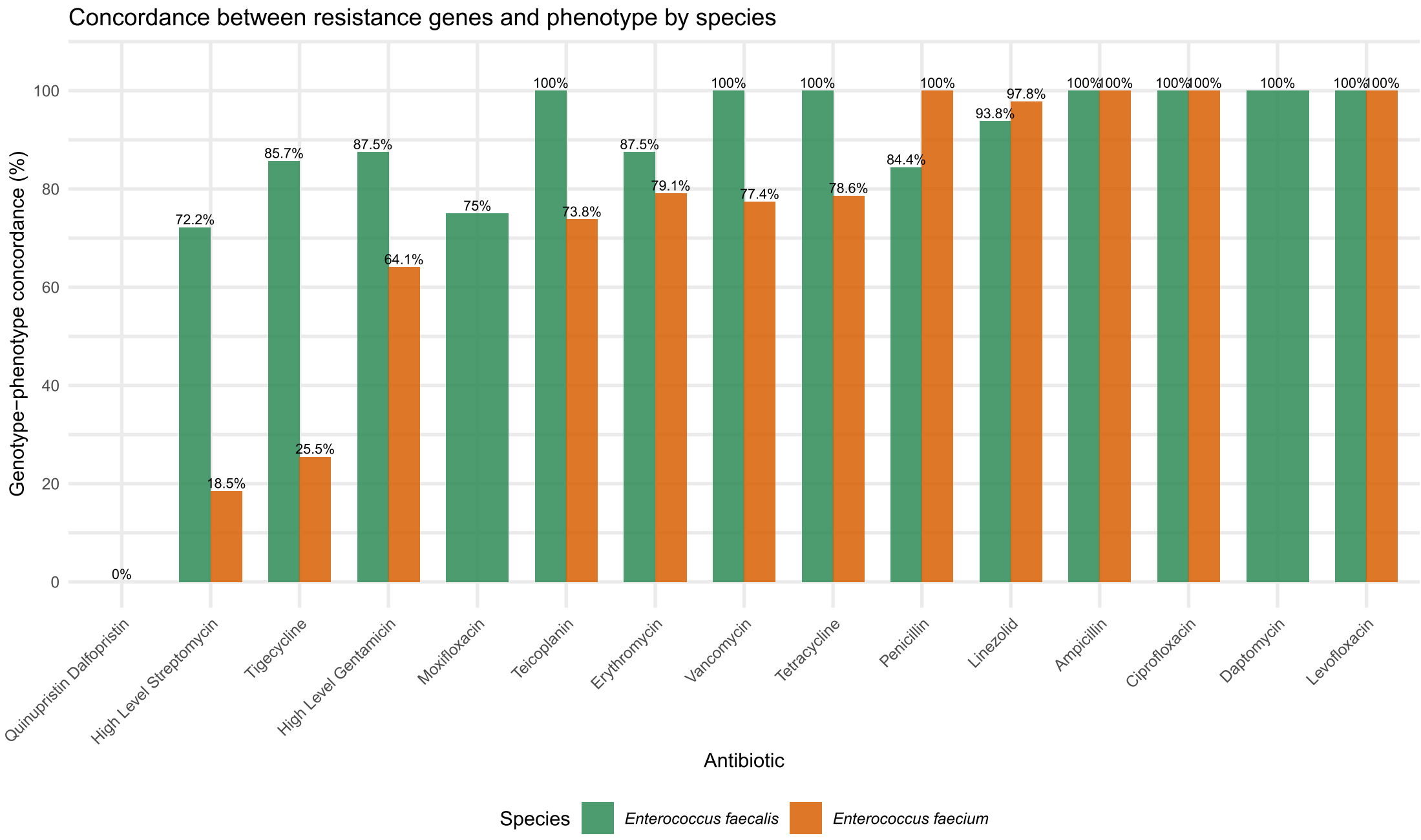

Figure S3. Concordance between genotypic and phenotypic resistance for each antibiotic by species. Bars represent the percentage of isolates in which the presence/absence of resistance genes (ResFinder) matched the phenotypic resistance category (R/S).

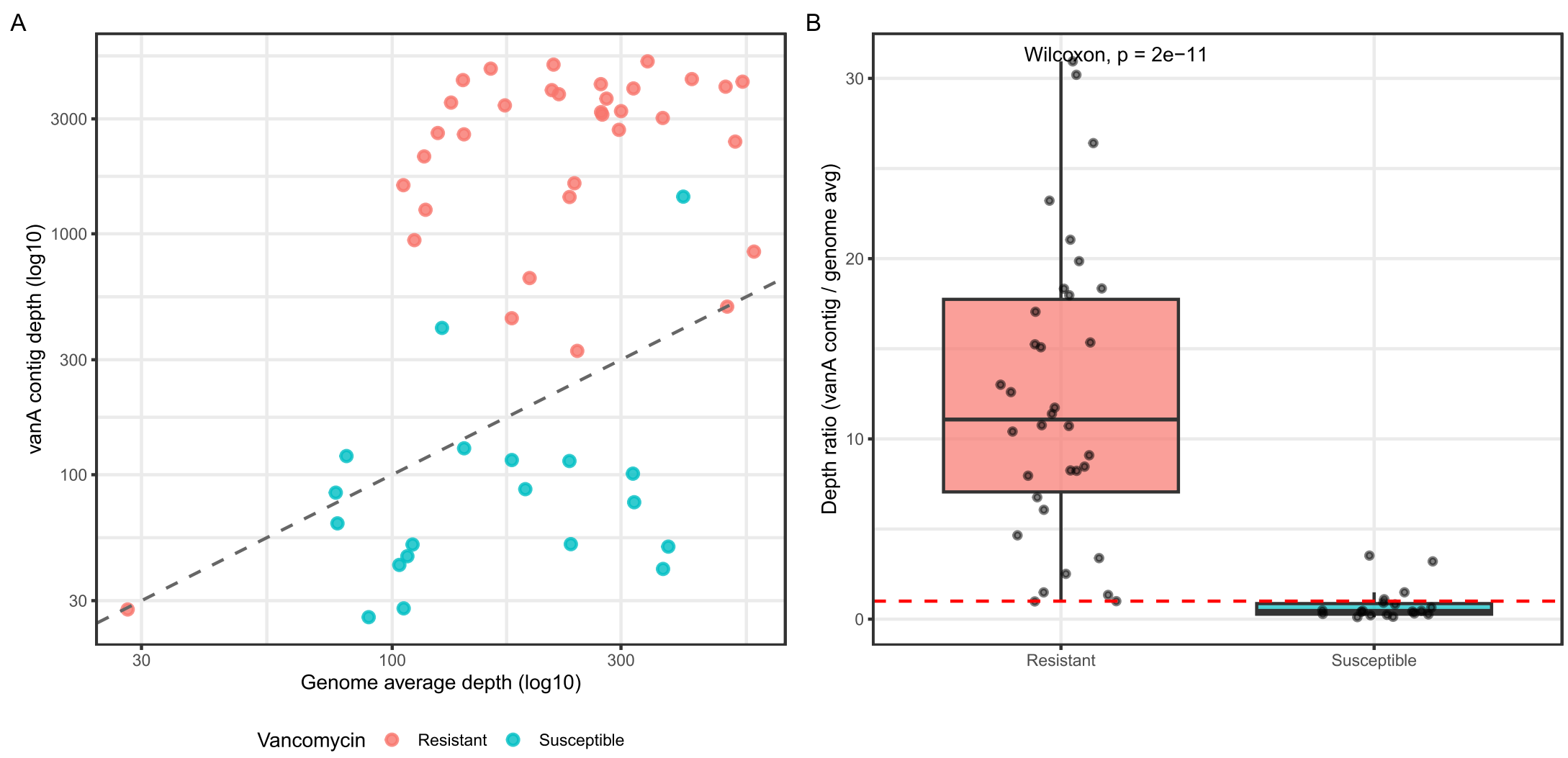

Figure S4. Relationship between *vanHAX* contig depth and genome-wide average depth in vanA-positive enterococcal isolates. (A) Scatter plot of *vanHAX* contig depth against genome average depth; the dashed line represents y = x (both axes log10-transformed). Colors indicate vancomycin phenotype (resistant or susceptible). (B) Box plot of depth ratios (*vanHAX* contig depth / genome average depth) for resistant and susceptible isolates. The horizontal dashed line marks a ratio of 1.

A

vanA gene tree

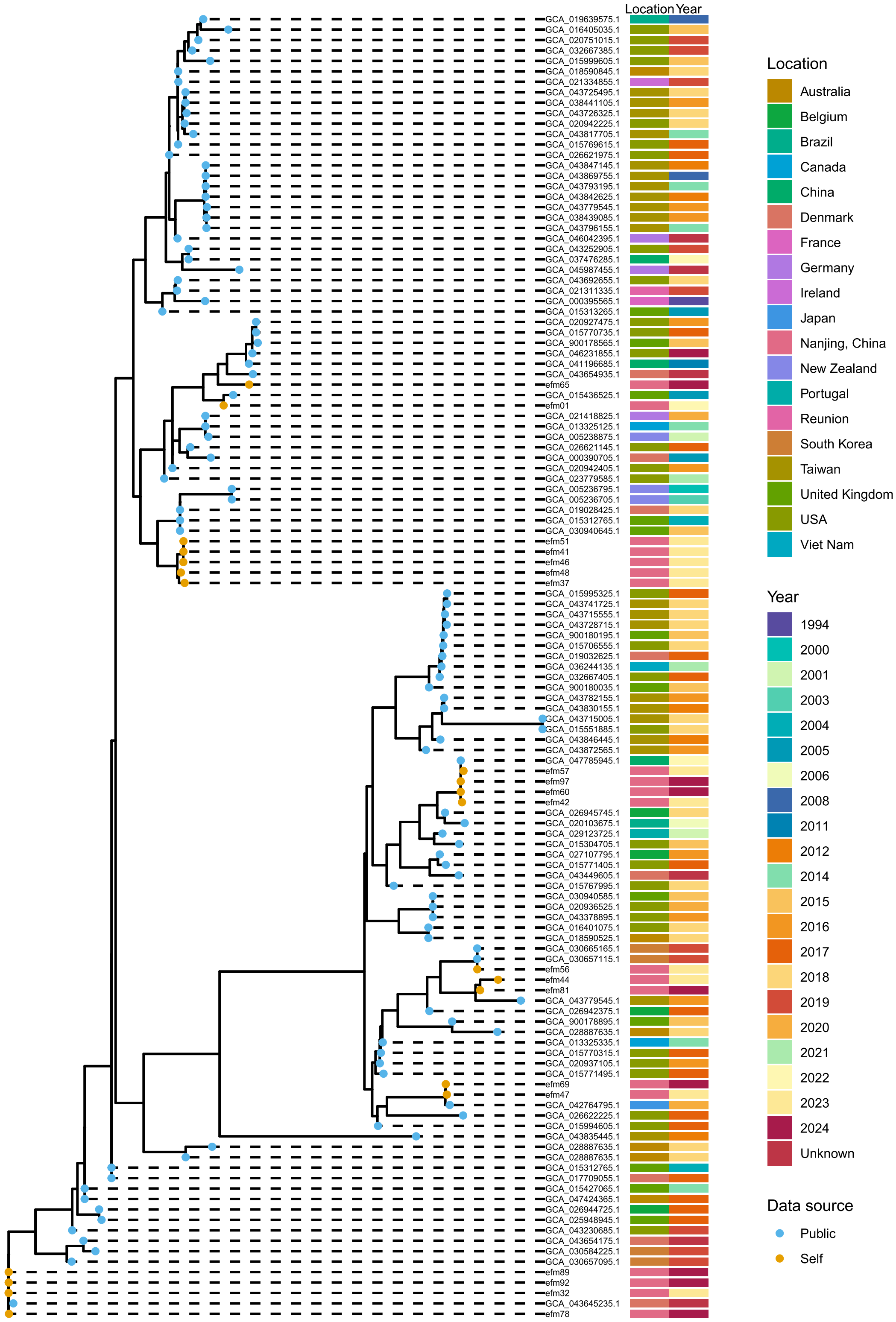

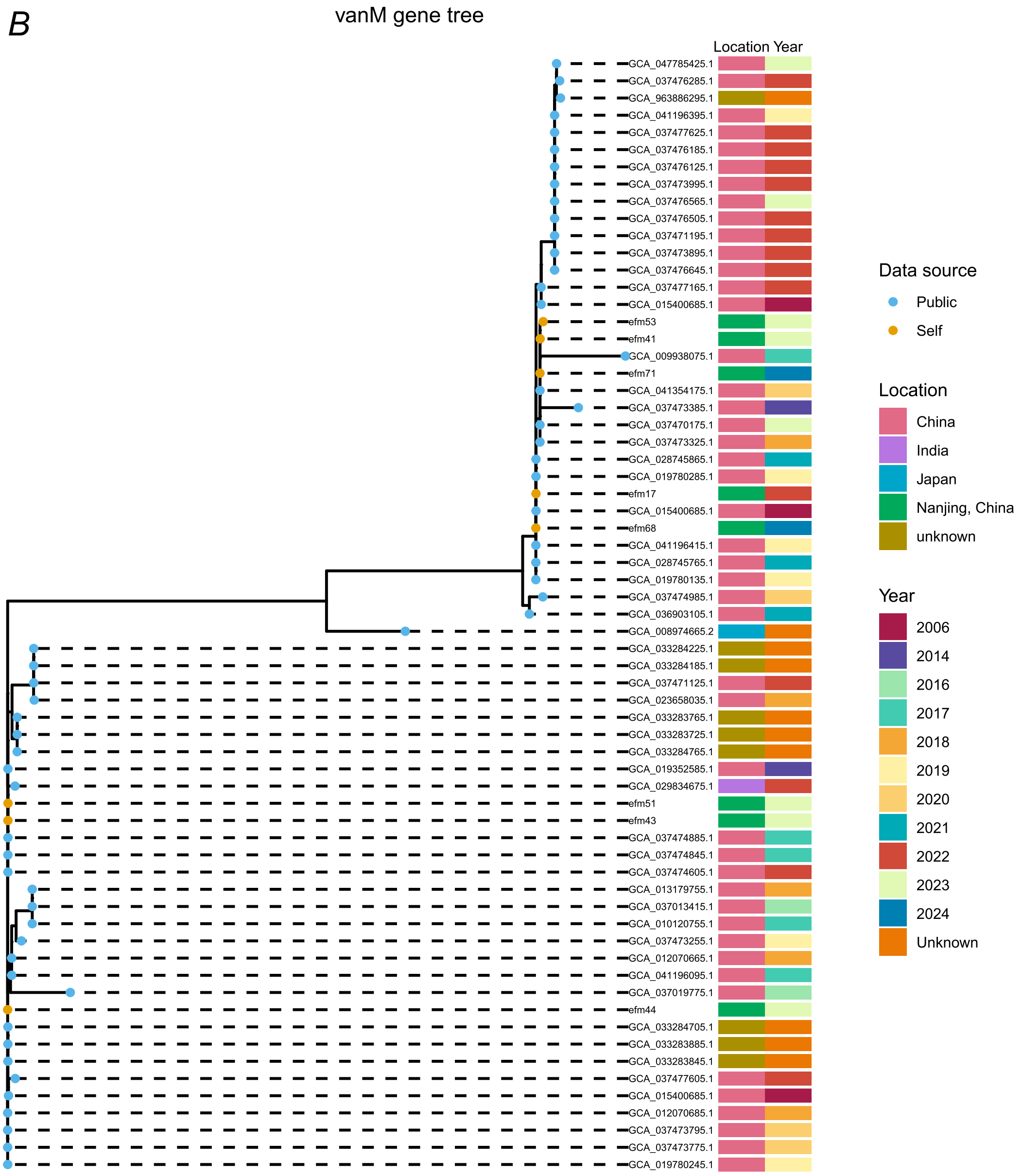

Figure S5. Phylogenetic analysis of *van*-associated transposon sequences. (A) Maximum-likelihood tree of *vanHAX* transposon regions; (B) maximum-likelihood tree of *vanHMX* transposon regions. Transposon boundaries were defined using a custom Python script that identified core *van* gene clusters (*vanH*, *vanA*/*vanM*, and *vanX* with intergenic distances <500 bp) and searched within a 15-kb flanking window for boundary markers, including transposase genes (IS elements, integrases, resolvases), regulatory genes (*vanR*/*vanS*), and accessory genes (*vanY*). The outermost coordinates of these markers defined the transposon ends, and the region was extended by 3,000 bp on both sides. For contigs lacking boundary markers within the 15-kb window, the region was defined solely by the core cluster plus 3-kb flanks. Trees were reconstructed from the core transposon regions using the maximum-likelihood method. Tip labels are colored by data source (self-sequenced isolates vs. public genomes). Additional annotation bars on the right indicate geographic origin and collection year. The resulting tree revealed that *vanHAX* sequences in our collection were closely related to epidemic strains from Taiwan (China), Japan, Denmark, and the United Kingdom, whereas *vanHMX* sequences were predominantly found in mainland China. Representative sequences are provided in Supplementary Table S6.
